# Sex-related differences in young binge drinkers on the neurophysiological response to stress in virtual reality

**DOI:** 10.3389/fpubh.2024.1348960

**Published:** 2024-06-14

**Authors:** Román D. Moreno-Fernández, Elena Bernabéu-Brotons, Myriam Carbonell-Colomer, Francisco Buades-Sitjar, Patricia Sampedro-Piquero

**Affiliations:** ^1^Facultad de Educación y Psicología, Universidad Francisco de Vitoria, Madrid, Spain; ^2^Departamento de Psicología Biológica y de la Salud, Facultad de Psicología, Universidad Autónoma de Madrid, Madrid, Spain

**Keywords:** alcohol, binge drinking, stress, neurophysiology, virtual reality, youth

## Abstract

**Background:**

Stress is one of the main environmental factors involved in the onset of different psychopathologies. In youth, stressful life events can trigger inappropriate and health-damaging behaviors, such as binge drinking. This behavior, in turn, can lead to long-lasting changes in the neurophysiological response to stress and the development of psychological disorders late in life, e.g., alcohol use disorder. Our aim was to analyze the pattern of neurophysiological responses triggered with the exposition to a stressful virtual environment in young binge drinkers.

**Methods:**

AUDIT-3 (third question from the full AUDIT) was used to detect binge drinking (BD) in our young sample (age 18–25 years). According to the score, participants were divided into control (CO) and BD group. Next, a standardized virtual reality (VR) scenario (Richie’s Plank) was used for triggering the stress response while measuring the following neurophysiological variables: brain electrical activity by electroencephalogram (EEG) and cortisol levels through saliva samples both measurements registered before and after the stressful situation. Besides, heart rate (HR) with a pulsometer and electrodermal response (EDA) through electrodes placed on fingers were analyzed before, during and after the VR task.

**Results:**

Regarding the behavior assessed during the VR task, BD group spent significantly less amount of time walking forward the table and a tendency toward more time walking backwards. There was no statistically significant difference between the BD and the CO group regarding time looking down, but when we controlled the variable sex, the BD women group displayed higher amount of time looking down than the rest of the groups. Neurophysiological measurements revealed that there was not any statistically significant difference between groups in any of the EEG registered measures, EDA response and cortisol levels. Sex-related differences were found in HR response to VR scenario, in which BD women displayed the highest peak of response to the stressor. Also, the change in heartbeat was higher in BD women than men.

**Conclusion:**

Unveiling the neurophysiological alterations associated with BD can help us to prevent and detect early onset of alcohol use disorder. Also, from our data we conclude that participants’ sex can modulate some stress responses, especially when unhealthy behaviors such as BD are present. Nevertheless, the moment of registration of the neurophysiological variables respect to the stressor seems to be a crucial variable.

## Introduction

Excessive alcohol intake is considered the most pervasive harmful habit in the world and has been identified as a social problem (1), causing 5.3% of all deaths worldwide (3 million deaths annually) according to the World Health Organization (WHO). This percentage rises among younger people (20–39 years of age), accounting for 13.5% of all fatalities in this age group, besides the effect on public health that this entails (2).

In recent years, college students have started to be considered a risk population for problematic alcohol use (3–5). The university stage is a time when the student builds a new social network and creates the foundation of their adult identity (6). Since most young people use alcohol as a recreational substance, alcohol use is quite prevalent at this period of life (7, 8). Additionally, the beginning of university studies also marks the onset of the legal drinking age, which allows them easy access and purchase of alcohol. A frequent pattern of alcohol consumption among young people is characterized by episodes of intense and compulsive drinking over a short period of time which is known as binge drinking (BD) and it has been correlated with high-risk behavior, such as aggression, reckless driving, or unprotected sex (9, 10). This represents a harmful alcohol-consumption pattern, and people who engage in this behavior may experience a variety of short- and long-term impacts, such as issues with their physical and mental health, violent behavior, or difficulty adjusting to family or school life (11, 12). Although quantitative definitions of BD differ significantly among nations, the most common threshold for defining BD is the intake of four to five alcohol doses (for men and women, respectively), in 2 h, at least once per month over a period of 6 months (13).

There is a great need for identification of biological markers associated with early alcohol-related brain damage. Concerning to this, BD is known to alter the frontal pathways (fronto-limbic) of white matter in adolescents and young adults (14, 15). The connections between the prelimbic and infralimbic regions, as well as those between the infralimbic region and the orbitofrontal cortex, appear to be affected by this diminished fronto-limbic connectivity (16). Besides, reduced or compromised inhibitory control seems to be associated with this decline in fronto-striatal connection (17). On the other hand, high alcohol consumption in university students has also been linked to a loss of gray matter in the prefrontal areas (18, 19) and cholinergic and serotonergic neurons persisting into adulthood (20). However, little is known about the impact of BD on physiological stress-related responses despite the relevance of this factor on alcohol abuse (21, 22). Some studies have pointed that young people and adolescents may be motivated to drink alcohol by their desire for new experiences and feelings, but, in general, they frequently drink alcohol to cope with the stress or unpleasant feelings they may have throughout their first year of college (due to the perception of responsibility, greater freedom, study, work, or the creation of new social networks) (23, 24). In comparison to the general population, university students often exhibit higher levels of anxiety and worst mental health (25, 26), and the primary source of this distress appears to be anxiety about academic success (27). Thus, lack of adequate strategies to deal with situations of stress can lead to the adoption of inappropriate or harmful coping mechanisms, such as excessive alcohol use.

Emotions include a psychophysiological component that can be studied using measurements of skin conductance (SC) and heart rate indices (HR) (28). SC is mainly influenced by the amount of moisture produced by the eccrine sweat glands under the control of the sympathetic branch of the autonomic nervous system (ANS) (29). HR is regulated by both the sympathetic and parasympathetic branches of the ANS, and it reflects stress reactivity in psychosocial and cognitive circumstances (30). Salivary cortisol is also frequently utilized as a biomarker of anxiety, and salivary cortisol levels seem an accurate indicator of the hypothalamus—pituitary–adrenal axis (HPA) response to stress (31). In summary, these three peripheral responses (SC, HR, and level of cortisol) are frequently used as measures of ANS reactivity (32) and, additionally, they are useful measurements for examining how drinking is related to emotional functioning (33, 34). Physiological arousal levels have been positively correlated with dependence and alcohol consumption levels (35), and gender differences in these responses also have been reported (36, 37).

In recent years, new research approaches have explored more ecological contexts and reliable measurements of stress-related responses. In this context, virtual reality (VR) has been increasingly employed because it is able to induce stress reactivity despite the virtual context (38). Currently, there is a dearth of studies that have investigated stress reactivity and anxiety-related behaviors in young individuals with substance use through exposure to virtual scenarios, being specially focus on treatment, craving or cognition (39–41). In a previous study of our group with young people with problematic alcohol consumption, without BD, we observed more anxious-related behaviors when they were exposed to our virtual environment, as well as a different pattern of physiological responses compared to the healthy youths (42). In consequence, we are interested in analyzing similar physiological responses when alcohol is consumed in BD way.

Therefore, our main goal was to analyze the physiological responses induced by a stressful VR environment in young people with BD. Along with this, we will consider the influence of participants’ sex in our results owing to both stress reactivity and regulation have been shown to differ between men and women, despite the neural networks supporting these processes remain unclear (43). We expect that these variables can constitute possible biomarkers that allow us to detect young people at risk of developing alcohol use disorder in the future.

## Methods

### Study sample

Volunteers were recruited from Francisco Vitoria University and Autonomous University of Madrid. Subjects included in the study signed the informed consent, accompanied by an informative note, and they created an alphanumeric code based on the first letter of their first name, the last letter of your first surname followed by your date of birth (e.g., PO1304) to guarantee privacy during the data processing and analysis phases. To collect information about alcohol use, anxiety trait, and depression symptoms on-line standardized questionnaires were administered using Qualtrics XM (Qualtrics, Provo, UT) by snowball sampling [Alcohol use disorders identification test (AUDIT, Spanish adaptation: (44))]; State–Trait Anxiety Inventory [STAI-t (45)]; Beck Depression Inventory-II [BDI-II (46)] (Table 1). Based on AUDIT-3 scores (“How often do you have 6 or more drinks on one occasion?”), we divided the sample into 2 groups: control group [CO, *n* = 23 (12 men and 11 women)]; BD group [*n* = 20 (8 men and 12 women)]. Inclusion criteria were: (1) aged 18–25 years; (2) an absence of diagnosis of substance use disorder; (3) an absence of comorbid disease, such as anxiety-related disorders, depression, psychotic disorder, or Attention-Deficit/Hyperactivity-Disorder (ADHD). Moreover, healthy controls should not present a history of drug abuse, including nicotine and alcohol. All volunteers were excluded if they presented severe difficulties in understanding the test instructions, altered consciousness or agitation, presence of fear of heights or claustrophobia, a score in the AUDIT above 19 points and if they consumed prescription drugs affecting the central nervous system (mainly anxiolytics and antidepressants). Additionally, during the initial interview, we asked them about possible phobias and individuals with fear of heights or claustrophobia were excluded from the study (Table 1). After the online assessment, a representative subgroup of participants was contacted by email to manage the VR session. In the case that some participant informed of nausea, dizziness, or blurred vision during the VR session, we would stop the session and he/she would be excluded from the study.

**Table 1:** Descriptive results for sociodemographic and emotional-related variables.

	CO		BD	
	Men	Women	Men	Women
Age	22.08 ± 0.38	20.91 ± 0.71	23.63 ± 0.38	20.33 ± 0.70
AUDIT	4.83 ± 1.46	5.45 ± 1.86	5.62 ± 0.99	11.67 ± 2.23
AUDIT-3	0.00 ± 0.00	0.00 ± 0.00	1.50 ± 0.19	1.58 ± 0.23
STAI trait	26.67 ± 0.97	26.36 ± 1.11	26.38 ± 1.89	28.91 ± 2.00
BDI-II	7.58 ± 1.60	9.63 ± 2.41	6.25 ± 1.58	11.00 ± 2.87
Fear of heights	1.82 ± 0.35	0.91 ± 0.21	1.00 ± 0.33	1.67 ± 0.36
Fear of spiders	1.18 ± 0.33	0.73 ± 0.19	0.88 ± 0.40	1.25 ± 0.37
VR experience	0.64 ± 0.20	0.73 ± 0.23	1.00 ± 0.19	0.58 ± 0.23

Values are mean ± SEM.

The study received approval from the Ethics Committee of the Autonomous University of Madrid (Code: CEI-122-2490) and Universidad Francisco de Vitoria (UFV 23-2022) in accordance with the Ethical Principles for Medical Research Involving Human Subjects adopted in the Declaration of Helsinki by the World Medical Association (64th WMA General Assembly, Fortaleza, Brazil, October 2013), Recommendation No. R (97) 5 of the Committee of Ministers to Member States on the Protection of Medical Data (1997), and the Spanish Data Protection Act (Ley Orgánica 15/1999 de Protección de Datos, LOPD). All participants were informed about the study prior to their inclusion in the study and then provided their written informed consent.

### VR setup

Participants received a VR headset (Meta Quest 2, 128 Gb, Meta Platforms Technologies Ireland Ltd.) and after checking the vision of participants, our VR EPM started. The immersive VR scenario chosen was Richie’s Plank on Oculus Quest 2, used successfully in previous research for evoking both stress response (47) and brain activity (47–49). Richie’s Plank Experience steam game is a VR game that makes the participant enter an elevator, takes them to the top floor of a skyscraper, and allows them to walk over and finally jump off a plank extending over the edge of the building. To increase the sensorial experience of the plank, we used a real wooden plank with 50 cm in diameter, the height 15 cm, and the length 160 cm. The VR scenario lasted approximately 5 min with 4 subsequent levels combining innate stressors, such as heights and spiders. We requested the consent of the participants to record their performance inside the task by casting the experience to a laptop. Behavioral variables registered were the following: percentage of time spent walking forward and backward, latency to walk forward (s) and time spent looking down in the edge of the plank (s). Behavioral variables registered were analyzed manually on the VR recordings by Raton Time program (42). Also, the different measures were taken by two independent observers and inter-rater reliability was determined by correlation analysis higher than 0.9.

### Psychophysiological measurements

EEG data were collected before (T1) and after (T2) the VR experience using Bitbrain’s Versatile 32 cap (model E32.A1), which is comprised of 32 electrodes (plus ground and reference) set in the international 10–10 system (Bitbrain Technologies 2018, Zaragoza, Spain). After informing them of what the task entailed, participants were seated in a comfortable chair during the EEG set-up, where the experimenter placed the EEG cap and ensured that all electrode impedances were kept below 5 Ω. Brain electrical activity was registered before and after the VR stressful situation [5 min eyes open (EO) and 5 min eyes closed (EC)] while staring at a fixation dot painted on the wall 90 cm from them. The conditions were counterbalanced so that half of the participants started with the EC condition and the other half started with the EO condition. The EEG signals were preprocessed using a combination of manual and automated procedures. First, all EEG recordings were visually examined by one of the authors in order to reject the most notable portions of noisy data. Then, the EEGlab (50) pipeline provided by Hatlestad-Hall et al. (51) was used to automatically reject the more subtle instances of noise in the data. This pipeline starts by iteratively referencing the data, searching for noisy channels, and excluding them from the computation of the average reference, which increases the robustness of the referenced data. Afterwards, a 1 Hz high-pass filter is applied, along with the removal of the power line noise using the nt_zapline function from the NoiseTools toolbox (52). Then, muscle and eye artifacts are identified using the SOBI algorithm (53) and subsequently removed using the IClabel function (54). Next, bad channels are interpolated, and a 40 Hz low-pass filter is applied. Finally, the continuous data is segmented into non-overlapping 4 s epochs for posterior analysis, and the two runs of the EO and EC conditions are, respectively, appended.

HR was registered with a pulsometer (Polar H10^®^) and EDA response through electrodes placed on fingers (eSense Skin Response^®^) before (T1), during (T2) and after (T3) the VR experience. Saliva samples were recollected (12 p.m. to 18 p.m.) through salivettes (Salivette^®^, Sarstedt, S.A., Barcelona, Spain) to analyze cortisol response before the start of the VR task (T1) and immediately after the end (T2). Saliva samples were coded and stored in the refrigerator at −20°C. Then, they were centrifuged at 1,500 rpm for 15 min, resulting in a clear supernatant of low viscosity that was stored at −80°C until the analysis of the salivary cortisol response (sCORT). Salivary cortisol levels were measured by an enzyme immunoassay kit (functional sensitivity 0.018 μg/dL) following the manufacturer’s instructions (Salimetrics, Stage College, PA, United States) (42).

### Statistical analysis

Statistical analyses were performed using SPSS 25 (IBM SPSS Statistics, Corporate headquarters, New Orchard Road, Armonk, New York 10504-1722, United States). All *p* values were two-tailed, and the level of significance was taken as *p* ≤ 0.05. Descriptive analyses (mean ± SEM) were performed on the demographic variables, information related to drug use and online questionnaires (more details are displayed in Table 1). GROUP (CO/BD) and SEX (men/women) were considered between-factors with two levels each and then, two-way repeated measures ANOVA (comparing T1/T2/T3) was performed for all the measures taken throughout the study (EEG, HR, EDA and salivary cortisol). Appropriate *post hoc* comparisons were conducted when significant differences were found. Additionally, given the small sample size, together with *p* value above mentioned (*p* ≤ 0.05), the critical value of each statistical analysis was used as the decision criterion, calculated *Post hoc* with G*Power based on the number of groups and number of measurements (55, 56).

## Results

### Behavioral results

**Figure 1:**
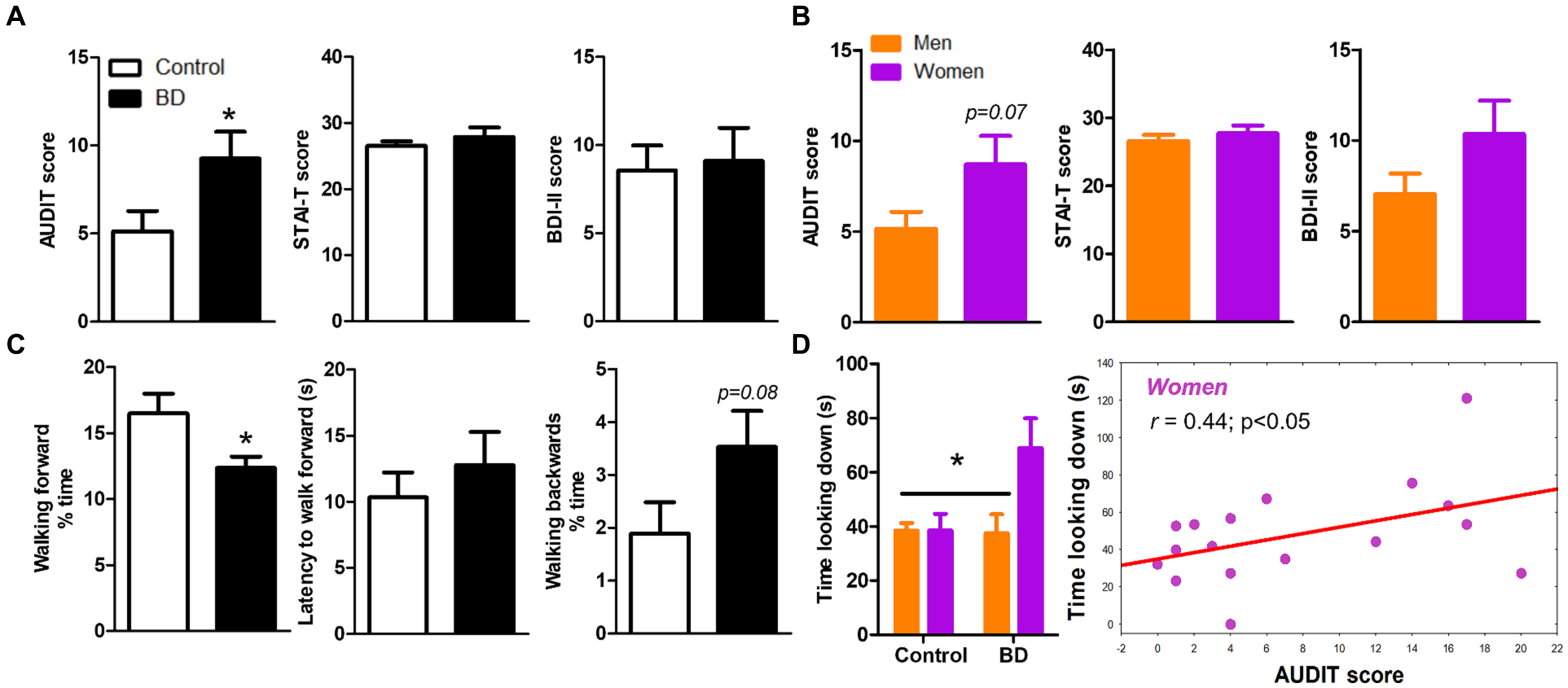
Psychological and behavioral variables of sample. **(A)** Means of AUDIT, STAI-T and BDI-II scores among groups (control and BD). **(B)** Scores in the same questionnaires separating men and women. **(C)** Behavior analysis in VR task (time walking forward, latency to walk forward and time walking backwards). **(D)** BD women group displayed higher amount of time looking down and this index correlates positively with AUDIT score.

First, with concern to the scores in the different tests administered, we observed no difference in STAI-T and BDI-II scores between groups (neither control vs. BD nor men vs. women; Figures 1A,B), but statistically significant high values in AUDIT scores in BD group [*t* (41)= − 2.19, *p* = 0.03, critical *t* = 1.64; Figure 1A] and a tendency toward significance in women [*t* (41) = 1.86; *p* = 0.07; critical *t* = 1.64; Figure 1B]. Regarding the behavior assessed during the VR task (Figure 1C), we observed that the BD group spent significant less amount of time walking forward the table [*t* (32)=2.44; *p* = 0.02] and a tendency toward more time walking backwards [*t* (32)= −1.79; *p* = 0.08; critical *t* = 1.44; Figure 1C]. There was no statistically significant difference between the BD and the control group regarding time looking down, but when we included gender, the BD women group displayed higher amount of time looking down than the rest of the groups [Group × Sex effect: *F* (1, 28) = 4.29, *p* = 0.04; critical *F* = 2.85; Figure 1D]. Interestingly, we found a significant positive correlation between AUDIT scores and time looking down in women group (*r* = 0.44; *p =* 0.03; Figure 1D).

### Physiological results

**Figure 2:**
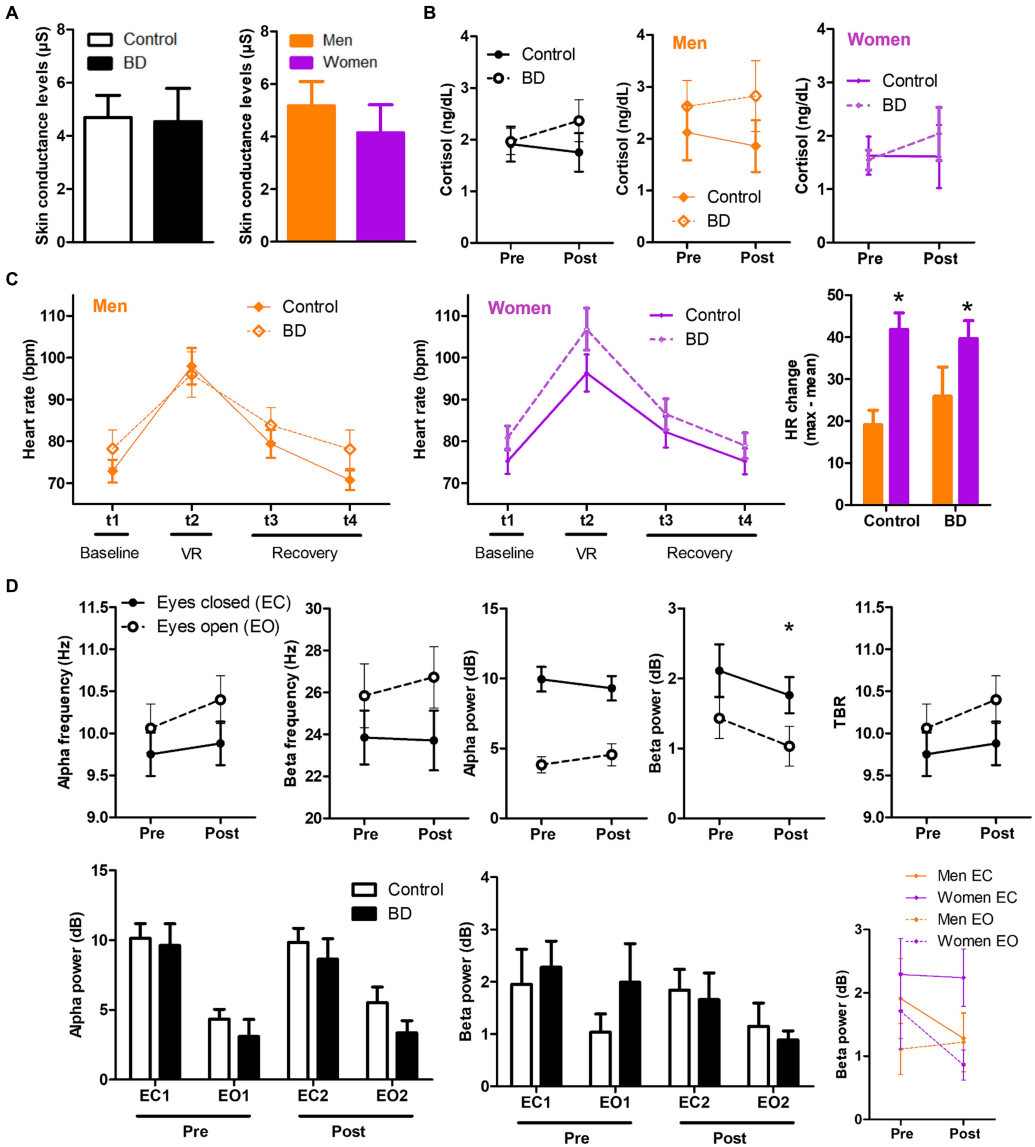
Sex-related differences in physiological variables measured in BD and control groups. **(A)** Skin conductance during the task. **(B)** Salivary cortisol levels before and after the VR task. **(C)** Interaction effect between group, time and sex in heart rate along the different measures (before, during and after VR stressor). **(D)** EEG parameters analyzed previous and after the stressful situation induced by VR.

With concern to the physiological response to VR stress, we found mixed results in different areas, all compiled in Figure 2. First, we did not observe any significant difference in the EDA response during the task when comparing for group or sex (Figure 2A). Secondly, there was no significant change of VR task in sCORT in the two measures taken (before the task and immediately after, Figure 2B). We did observe a significant effect of time in heart rate parameter. In fact, repeated-measures ANOVA revealed significant sex-related changes in HR response to VR [time × group × sex, *F* (3, 117) = 3.86; *p* = 0.01; critical *F* = 2.68; Figure 2C], in which BD women displayed the highest peak of response to the stressor, and BD men showed lower response. Also, the change in heartbeat was higher in women than men (sex effect; Figure 2C).

Finally, the differential EEG signal between BD and CO group was analyzed before and after the VR. At this point, there was not any statistically significant difference between groups in any of the EEG measures determined (Figure 2D). However, we observed significant differences between T1 and T2 in beta power (dB) of both EEG conditions, eyes open (EO) and eyes closed (EC) (Figure 2D).

## Discussion

In the current study, we explored the behavioral and neuropsychophysiological responses of young individuals with BD during the exposition to a stressful VR environment compared to a healthy CO group. Our stress-related variables, registered at different moments of the VR task (behavioral pattern of ambulation, EEG, HR, and EDA response, as well as salivary cortisol), could be possible used as possible biomarkers that allow us to detect young people at risk of developing alcohol use disorder in the future, as well as anxiety-related disorders (57–59). As expected, our results showed several behavioral and physiological differences between groups, highlighting the variable sex as an important factor modulating our results.

Concerning the behavioral performance during the VR task, our results showed that the BD group spent significantly less time walking forward the table with respect to the CO group, whereas the time spent looking down was similar in both groups. Nevertheless, when we controlled the variable sex, the BD women group displayed higher amount of time looking down than BD men. As we know, this is the first study that has employed the software Richie’s Plank Experience to analyze the behavioral pattern of exploration of young participants with alcohol consumption in BD way. Studies in animal models (60, 61) and in humans (42, 62, 63) have shown that the higher the level of anxiety, the more we tend to avoid unprotected open spaces. Hence, our BD participants did not spend much of the experience walking the plank, let alone getting to the end of it. On the contrary, although it was not significant, they showed a tendency to walk backwards toward the protected space without turning around as an indicator of anxiety and fear of falling. Previous study of our group using another VR experience revealed similar results with a more anxious pattern of exploration in young subjects with problematic alcohol use (42). It results interesting that BD women displayed higher time looking down during the experience respect to BD men. This result could be understood in two ways. On the one hand, it is possible that the women from the experimental group exhibited more risk-taking and sensation seeking-related behavior, or on the other hand, this behavior could have reflected an attempt to check that they were not going to fall while walking slowly on the board. Regarding the first interpretation, some studies have observed that women in the mid- and late-20s usually, as in our study, usually show higher scores in sensation seeking behaviors (64, 65) which, in turn, is related to seek out novel and exciting stimuli, including risk-taking behavior (66). Further research on this matter is necessary due to the clinical implications that this variable presents as a risk factor (67).

EEG measurements were not different between groups, as well as EDA response and the salivary cortisol levels. It is possible that the lack of differences in salivary cortisol levels were due to methodological errors associated with the pattern of release of this hormone. We recollected the T2 sample immediately after the end of the VR task, whereas cortisol peak occurs between 20 and 30 min after stressor onset and can continue to be elevated for an hour or more (68, 69). A previous study of our group also found no differences in EDA response between young participants with problematic alcohol consumption and the CO group (42). Some studies have suggested that blunted reactivity is a general characteristic of alcohol dependency, particularly in adults with a long-standing history of consumption, rather than in individuals with time-limited excessive alcohol use as in our studies (70). Regarding EEG registered variables, recent literature has described that, similar to individuals with alcohol addiction, BD also displayed increased resting-state EEG signal (59). For instance, high-BD subjects presented greater spectral power over the frontal, central and parietal regions in the delta (0–4 Hz), and fast-beta (20–35 Hz) frequency bands, suggesting that high-BDs display an EEG spectral pattern similar to that observed in subjects with alcohol use disorder (70, 71). Similarly, Affan et al. (58) found a slower Alpha Peak Frequency as well as increased frontal theta and beta power in the BD group. Hence, resting-state EEG studies seem to indicate that BDs present abnormal spontaneous EEG signal, mainly characterized by increased power in slow (delta/theta) and fast (beta) frequency bands which could be associated with a brain overactivity eventually caused by an excitatory-inhibitory imbalance (72). In our study, the CO and BD group were similar in Alpha power which is not consistent with previous studies. However, reduced alpha power was observed mainly when anxiety symptoms are present in the BD sample (73, 74). This aspect was controlled in our study (see STAI and BDI-II results in Table 1) and could have contributed to the non-significant difference between groups. On the other hand, the short history of alcohol use in our BD sample could be also related to the lack of differences with the CO group (72, 75).

Sex-related differences were found in HR response to VR scenario, in which BD women displayed the highest peak of response to the stressor. Also, the change in heartbeat was higher in BD women than men. This enhanced HR response in BD women could be related to their risky behaviors during the exploration of the VR environment. Thus, it has been widely described that to cope with stressful stimuli our body releases adrenaline, a hormone that temporarily causes higher heart rate to speed up and your blood pressure to rise (76). Hence, it could be a normal biomarker of their pattern of ambulation during the VR environment. These findings are consistent with the theory that increased HR reflects engagement of self-regulatory efforts to cope with an emotional or stressful situation (77). Considering the historical under-representation of women in alcohol abuse research, the identification of HR as a potential biomarker for addiction and relapse risk (78), and the close relationship between stress, alcohol abuse, and relapse in women (79), these issues hold significant research and clinical relevance.

In general, VR is a technological tool which can open new avenues to better understand the neurobiological mechanisms of emotional responding in healthy and pathological conditions (42). It results attractive to young people and, while its employment is more prevalent in the intervention context, it could be employed as a tool to identify behavioral patterns or physiological responses related to future clinical conditions. Moreover, it is notorious to highlight the role of sex variable in addiction related studies in which girls usually show a tendency toward increased alcohol consumption associated with negative mood states (80). Additionally, their different psychophysiological responses to a stressor may be useful as a future predictor of severity. As limitations of our study, we would like to mention the reduced sample size. Moreover, variables such as the menstrual phase, as a variable influencing stress response or drug-related variables, as the quantity of alcohol consumed by the participants, should have been considered in our study.

## Conclusion

The present study focused on examining the behavioral signs of anxiety through a VR task and investigating the psychophysiological responses associated with a virtual stressful context. Exploring biomarkers provides crucial insights into the intricate relationship between the body and mind, and VR appears to be a useful tool for investigating anxiety in humans. Therefore, understanding and addressing the biological mechanisms involved in stress-related responses holds the promise of improving treatments, predicting problematic use, and provide personalized care to address the specific needs of women.

## Data availability statement

The raw data supporting the conclusions of this article will be made available by the authors, without undue reservation.

## Ethics statement

The study received approval from the Ethics Committee of the Autonomous University of Madrid (Code: CEI-122-2490) and Universidad Francisco de Vitoria (UFV 23-2022) in accordance with the Ethical Principles for Medical Research Involving Human Subjects adopted in the Declaration of Helsinki by the World Medical Association (64th WMA General Assembly, Fortaleza, Brazil, October 2013), Recommendation No. R (97) 5 of the Committee of Ministers to Member States on the Protection of Medical Data (1997), and the Spanish Data Protection Act (Ley Orgánica 15/1999 de Protección de Datos, LOPD). All participants were informed about the study prior to their inclusion in the study and then provided their written informed consent. The studies were conducted in accordance with the local legislation and institutional requirements. The participants provided their written informed consent to participate in this study.

## Author contributions

RM-F: Conceptualization, Formal analysis, Investigation, Methodology, Project administration, Supervision, Writing – original draft, Writing – review & editing. EB-B: Investigation, Writing – original draft. MC-C: Investigation, Methodology, Writing – original draft. FB-S: Formal analysis, Writing – original draft. PS-P: Conceptualization, Funding acquisition, Investigation, Methodology, Project administration, Writing – original draft, Writing – review & editing.
